# Rapid assessment of target-binding fractions in molecular imaging agents using size-exclusion HPLC

**DOI:** 10.1186/s41181-026-00469-5

**Published:** 2026-07-04

**Authors:** Ashtyn McAdoo, Kamal Jouad, Eben L. Rosenthal, Adam J. Rosenberg

**Affiliations:** 1 Chemical and Physical Biology, Vanderbilt University, Nashville, TN USA; 2 Department of Otolaryngology-Head and Neck Surgery, Vanderbilt University Medical Center, Nashville, TN USA; 3 Vanderbilt University Institute of Imaging Science, Vanderbilt University Medical Center, Nashville, TN USA; 4 Department of Radiology and Radiological Sciences, Vanderbilt University Medical Center, Nashville, TN USA

**Keywords:** Immunoreactivity, Target-binding fraction, Lindmo assay, Quality control, Radiochemistry, Radioimmunoconjugates

## Abstract

**Background:**

The clinical translation of molecularly targeted therapeutics and imaging agents represents a cornerstone of precision oncology, with the global theranostics market projected to exceed $25 billion by 2030. However, the development of theragnostic agents or diagnostic companions remains constrained by analytical bottlenecks in quality control, such as target-binding fraction, which are increasingly required by regulatory agencies as product release criteria during the translation process. Current methods, including enzyme-linked immunosorbent assay (ELISA), which require specialized resources or external CROs, or bead-based assays for radiolabeled compounds, which involve complex multi-step protocols; these limitations and others hamper their practical implementation in clinical manufacturing environments. Assay delays can postpone clinical trial initiation, increase development costs, and delay patient access to these agents.

**Results:**

We have developed and validated a rapid, size-exclusion high-performance liquid chromatography (SE-HPLC) method for the determination of target-binding fractions of labeled biologics. The method separates the unbound biologic from the larger antigen-bound complex, allowing for rapid quantification. We validated the method using a panel of fluorescently labeled antibodies (panitumumab-IRDye800CW, nivolumab-IRDye800CW) and radiolabeled biologics ([18F]GEH200521, [18F]NOTA-ABY-030), assessing linearity, specificity, and concentration independence. The SE-HPLC method achieved excellent separation of bound and unbound species with a resolution (Rs) of 3.2. A strong linear relationship (R^2^ = 0.999) was observed between the antigen-to-antibody ratio and the measured binding fraction. The method demonstrated high specificity, with no binding detected with non-target antigens. The total assay and analysis time was less than 35 min, a significant improvement over traditional methods.

**Conclusions:**

SE-HPLC provides a rapid, specific, and cost-effective alternative to traditional binding fraction assessment methods, reducing quality control timelines from weeks/hours to minutes. The method’s compatibility with both fluorescent and radiolabeled biologics and integration with existing HPLC infrastructure represents a significant advancement in development workflows.

**Supplementary Information:**

The online version contains supplementary material available at https://doi.org/10.1186/s41181-026-00469-5.

## Background

The era of precision oncology has been defined by the development of molecularly targeted therapeutics, which have transformed cancer treatment paradigms (Min and Lee 2022; Lheureux et al. 2017; Renfro et al. 2017; Orlova et al. 2006; Löfblom et al. 2010; Day et al. 2013; Martelli et al. 2016; Solomon et al. 2011; Kircher et al. 2012; Giuliani et al. 2024; Stupp et al. 2005; Brahmer et al. 2023). The global market for these agents, including antibody–drug conjugates, immune checkpoint inhibitors, and radiolabeled theranostics, is projected to exceed $100 billion in 2024 (McAdoo et al. 2025). The clinical success of these biologics is contingent upon their ability to bind to their intended molecular targets with high specificity and affinity. Therefore, a critical component of the manufacturing process is the analytical measure of the target-binding fraction/potency, a key quality attribute that is mandated by regulatory agencies (Center for Drug Evaluation and Research and Center for Biologics Evaluation and Research 2015). The clinical success of targeted therapeutics is dependent on the interaction between the targeting vector and its intended target. These therapeutics require analytical validation to ensure that the targeting vector maintains its binding potency throughout the manufacturing process. When these biologics are conjugated with imaging or therapeutic labels, additional analytical validation is required to ensure the labeling process does not alter target-binding integrity. The binding fraction (the percentage of labeled biologic that retains the ability to bind its intended target fraction) serves as a critical quality measure that correlates with therapeutic efficacy and patient safety. The regulatory framework for targeted biologics mandates rigorous assessment of binding potency throughout the manufacturing process, for patient safety and efficacy (Center for Drug Evaluation and Research and Center for Biologics Evaluation and Research 2015). The United States Food and Drug Administration (FDA), European Medicines Agency (EMA), and other regulatory authorities require reproducible analytical validation demonstrating that labeled biologics maintain their target-binding properties throughout the manufacturing process and product lifecycle (Center for Biologics Evaluation and Research and Center for Drug Evaluation and Research 1997; Delaney et al. 2024; Rhodes et al. 1990).

To assess the functional integrity of the labeled biologic, reactivity assays like ELISAs involve multiple incubation, blocking, and washing steps that are labor-intensive and time-consuming (Hornbeck 2015). The Lindmo assay, widely used for radiolabeled compounds, relies on cell-based systems requiring specialized cell culture facilities, trained personnel, and extensive preparation time (Lindmo et al. 1984; Mattes 1995; Dux et al. 1991). More recently developed bead-based assays, while offering some improvements in throughput, still involve multi-step protocols and specialized equipment that limit their practical implementation (Sharma et al. 2019).

High-performance liquid chromatography (HPLC) represents a mature, widely available analytical platform that is already integral to the quality control of biologics manufacturing (Lam et al. 2025; Fekete et al. 2014) When a targeting biologic binds to its intended antigen, the resulting complex has a substantially larger molecular weight than the unbound biologic, enabling chromatographic separation (Zamora et al. 1994). Previously, researchers at Genentech developed an HPLC-based assay to measure the target-binding fraction of a zirconium-89-labeled minibody (Kist de Ruijter et al. 2022). Here we describe the development, validation, and clinical implementation of a size-exclusion HPLC method for rapid assessment of target-binding fractions in both fluorescently labeled and radiolabeled biologics.

## Results

### Chromatographic method optimization and column performance evaluation

To establish an HPLC-based quantification of the target-binding fraction, we evaluated two size-exclusion chromatography (SEC) columns. Initial assessment using a Biozen 1.8 μm dSEC-2 column achieved adequate separation of panitumumab-IRDye800CW from its EGFR complex, with retention times of 7.2 ± 0.1 min for the unbound antibody and 5.7 ± 0.1 min for the antibody-antigen complex, respectively. While the peak symmetry was excellent (tailing factors < 1.2), the resolution (Rs) was 1.8, which provided a limited margin for method robustness.

**Fig. 1:**
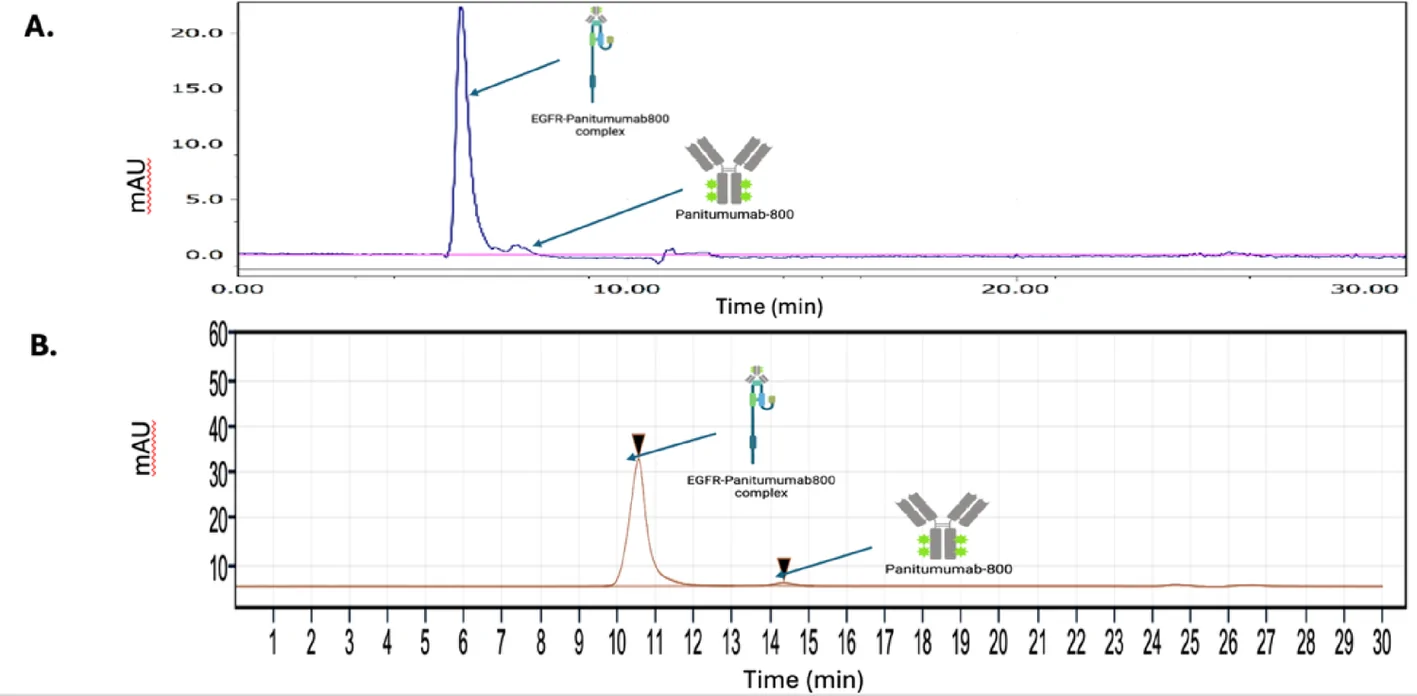
Representative chromatograms of panitumumab-IRDye800CW incubated with and without EGFR, showing the separation of the antibody-antigen complex from the unbound antibody on the **A** Biozen 1.8 μm dSEC-2 and **B** Superdex 200 column. The high resolution (Rs = 3.2) allows for accurate quantification of both species

In contrast, the Superdex 200 Increase 10/300 GL column demonstrated superior separation performance. Using this column, the unbound panitumumab-IRDye800CW eluted at 14.1 ± 0.1 min, while the antibody-EGFR complex eluted at 10.4 ± 0.1 min, yielding an improved resolution of 3.2 (Figs. 1, S1, and S2). Based on this enhanced performance, the Superdex 200 column was selected for further evaluation.

### ELISA validation

To support the results for the SE-HPLC method, the binding affinity by ELISA for pan800 was measured. A sample was shipped to an established CRO (RayBioTech), where EGFR binding was evaluated using an established ELISA protocol (successful in prior IND applications). The Pan800 demonstrated binding potency (EC_50_) comparable to that of unconjugated panitumumab (PAN & Pan800 5 ± 2.5 ng/mL), and demonstrated that the lot of Pan800 was suitable to be used as a model compound. While ELISA measures the binding potency different from the target binding fraction assay, the two results are both derived from the binding region of the Pan800 remaining intact.

### Antigen to antibody ratio

**Fig. 2:**
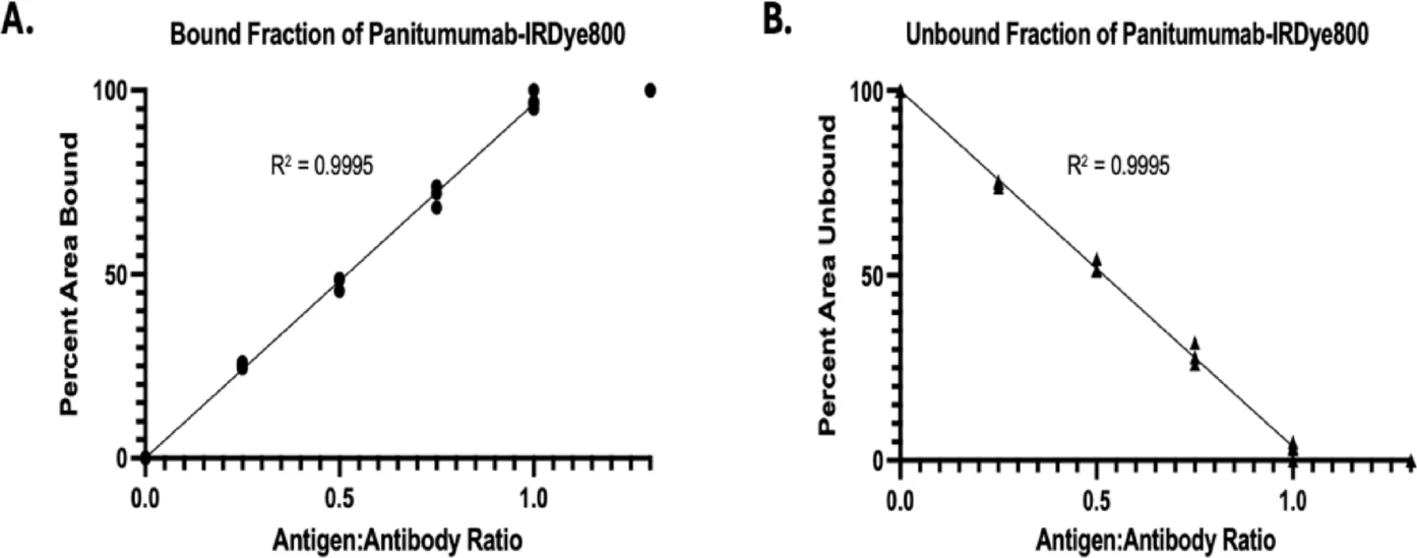
Linear regression analysis of the binding fraction of panitumumab-IRDye800CW as a function of the antigen-to-antibody ratio. The high correlation coefficient (R^2^ = 0.999) demonstrates the linearity of the method over the tested range

The linearity of the SE-HPLC method was assessed by analyzing samples with varying molar ratios of EGFR to panitumumab-IRDye800CW. While the antigen volume remained constant, increasing amounts of pan800 were added to the antigen solution and analyzed by HPLC to generate the antigen-to-antibody ratios tested. As shown in Fig. 2, the percent area under the curve for both the bound (10.4 ± 0.1 min) and the unbound (14.1 ± 0.1 min) peaks varied proportionally to the input ratio, confirming method linearity.

A linear regression was performed to assess the relationship between the antigen-to-antibody ratio (between the ratios 0 and 1) and the percent area bound or the percent area unbound (representing the labeled antibody) (Table 1). The R^2^ value for each graph is 0.999, indicating the linearity of the relationship between the amount of antibody bound or unbound and the ratio of antigen-to-antibody. At a 1.3:1 ratio, where the antigen was in excess, the antibody was 100% bound, confirming the saturation of the system. All experiments were conducted in triplicate to ensure reproducibility, and the results were consistent across all replicates. Linear regression analysis of the data from the 0.25:1 to 1:1 ratios demonstrated an excellent linear relationship (R^2^ = 0.999, *p* < 0.001). The slope of the regression line was 94.2 ± 1.1%, which is close to the theoretical value of 100%, and the y-intercept of − 1.8 ± 0.7% was not significantly different from zero (*p* = 0.052), indicating the absence of systematic bias in the method.

**Table 1:** The bound versus unbound peak areas at varying concentrations of panitumumab-IRDye800 using a 1:1 antigen-to-antibody ratio. Each concentration was run in triplicate

Panitumumab-IRDye800CW concentration (mg/mL)	Percent bound to antigen (%)	Percent unbound to antibody (%)
11.1	97.5 ± 0.8	2.5 ± 0.8
5.5	96.9 ± 3.1	6.2 ± 3.1
2.8	95.7 ± 0.7	4.3 ± 0.7
1.4	98.5 ± 1.5	1.5 ± 1.5
0.7	98.8 ± 0.7	1.2 ± 0.7
Average	97.4 ± 3.6	2.6 ± 3.6
RSD	3.7%	

### Assay specificity

Binding specificity was supported by negative control experiments, which demonstrated no detectable interaction with non-target antigens (Table 2). When panitumumab-IRDye800CW was incubated with non-target antigens HER2 and PD-L1, no binding was detected, with 100% of the fluorescent signal remaining in the unbound fraction. Similarly, when EGFR was incubated with non-target antibodies, including the anti-amyloid antibody lecanemab-IRDye800CW and an isotype control antibody (IgG2-IRDye800CW), no binding was observed (Table 2).

**Table 2:** Negative controls of both the antibody and antigen were used to ensure specificity of the antibody, panitumumab-IRDye800, to the antigen, EGFR

Antibody	Antigen	Percent bound to antigen (%)	Percent unbound to antibody (%)
Panitumumab-IRDye800	EGFR	97.5 ± 0.8	2.5 ± 0.8
lgG-IRDye800	EGFR	0 ± 0	100 ± 0
Lecanumab-lRDye800	EGFR	0 ± 0	100 ± 0
Panitumumab-lRDye800	HER2	0 ± 0	100 ± 0
Panitumumab-lRDye800	PD-L1	0 ± 0	100 ± 0

### Validation of the assay with additional fluorescent and radiolabeled biologics

To demonstrate the broad applicability of the SE-HPLC method, we tested it with a range of other fluorescent and radiolabeled biologics.

Nivolumab-IRDye800, an anti-PD-1 antibody, was incubated with its target antigen, PD-1. The resulting complex eluted at 12.2 ± 0.1 min, while the unbound antibody eluted at 14.3 ± 0.1 min, similar to the panitumumab system (Figs. S3 and S4). The analysis showed that 100% of the nivolumab-IRDye800 was bound to PD-1, demonstrating the utility of the method for other fluorescently labeled antibodies.

### Validation in other targeted agents

The method was successfully applied to two different radiolabeled biologics. First, the immunoreactivity of [^18^F]GEH200521, a nanobody-based radiopharmaceutical targeting CD8, was assessed. Upon incubation with the CD8 antigen, the binding fraction was determined to be 97.55% ± 0.83% (Figs. S5–S8). Second, the binding of [^18^F]NOTA-ABY-030, an affibody-based radiopharmaceutical targeting EGFR, was quantified (Figs. S9 and S10). The analysis showed a binding fraction of 89.67% (standard deviation = 1.46). These results confirm that the SE-HPLC method is compatible with radiolabeled biologics and can provide rapid QC data for these time-sensitive agents.

Purity analysis of [^18^F]GEH200521 showed a primary retention peak at 8.28 ± 0.17 min, representing 92.04% ± 4.14% of the sample. Upon incubation with CD8 antigen, the resulting [^18^F]GEH200521–CD8 complex exhibited a shifted retention time of 5.57 ± 0.04 min, while the unbound [^18^F]GEH200521 peak shifted slightly to 8.63 ± 0.13 min. The complex comprised 87.33% ± 4.14% of the total area, and the unbound antibody accounted for 3.72% ± 1.06%. When considering only the peaks corresponding to the bound and unbound antibody, 97.55% ± 0.83% of [^18^F]GEH200521 was bound to CD8, with 2.45% ± 0.83% remaining unbound.

## Discussion

Because conventional methods of measuring binding specificity are costly and inefficient in a manufacturing environment, we compared these methods to size-exclusion HPLC. We demonstrated this using both fluorescently labeled therapeutic antibodies (Pan800, Nivo800) and two radiolabeled imaging agents, such as [^18^F]GEH200521 and [^18^F]NOYA-ABY-030. Our findings demonstrate that this method provides a robust and reliable alternative to traditional binding assays, addressing a critical bottleneck in the quality control (QC) pipeline for the clinical translation of these agents. Our findings suggest this as an alternative to more time-intensive cell or bead-based assays for use in quality control (QC) assessments of molecular imaging agents.

While the principle of using SE-HPLC to separate bound from unbound species based on molecular weight has been previously demonstrated to determine the immunoreactive fraction of ZED8, a ^89^Zr-labeled anti-CD8 antibody, for cGMP-compliant manufacturing (Gill et al. 2020; Kist de Ruijter 2022). The SE-HPLC and Lindmo cell-based assays were directly compared in Gill et al. (2020) with comparable results. While the ZED8 study focused on a single radiolabeled antibody, we have extended SE-HPLC assay to fluorescently labeled antibodies (panitumumab-IRDye800CW and nivolumab-IRDye800), a radiolabeled nanobody ([^18^F]GEH200521), and a radiolabeled affibody ([^18^F]NOTA-ABY-030). Here we demonstrate the value of this technique with a range of scaffold sizes and types of labels.

The linearity of the relationship between the amount of antibody (bound and unbound fractions) and the antigen-to-antibody ratio had an R^2^ value of 0.999. A 1:1 molar ratio of EGFR to panitumumab-IRDye800 was adequate for assessing the binding fraction of panitumumab-IRDye800, although complete saturation was not achieved, potentially due to experimental variability of the Pan800 and/or concentration. Although variability in the concentration of reactants can also lead to inconsistent results, this was not observed in the current study. When the concentration of pan800 was varied between 0.7 and 11.1 mg/mL, no significant differences were observed in the results, demonstrating consistency and reproducibility of the method across varying concentrations and productions of labeled panitumumab. This technique is especially relevant for radiolabeled biologics, where binding fraction detection is often performed at microgram to nanogram concentrations per milliliter. Furthermore, we had a total analysis time of under 35 min, which also benefits uses in radiolabeling, allowing a “same-day” release strategy.

Chromatographic separation may perturb the binding equilibrium; partial dissociation of ligand-target complexes may occur during analysis. As such, the measured binding fraction may be influenced by binding affinity, with optimal performance expected for high-affinity molecular probes such as antibodies. However, any dissociation of the probe/antigen complex on the HPLC will serve to decrease the measured target binding fraction resulting in a lower assay value. Future studies will evaluate the relationship between affinity and measured binding fraction to define the operational limits of the method. Despite these considerations, the method demonstrated consistent performance across the high-affinity systems evaluated in this study; which are thought to be reflective of the general high-affinity nature of imaging agents entering clinical trials.

Because the 280 nm channel detects all proteins, including both antibody and antigen, it is unsuitable for assessing binding specificity. In contrast, for optically labeled compounds, the 780 nm wavelength chromatogram enables selective detection of the labeled drug without interference from excess or unbound antigen. Likewise, the gamma detection channel offers comparable specificity for radiolabeled biologics, detecting only the radiolabeled compound. These channels allow for a more accurate evaluation of the labeled antibody’s capacity to bind its target antigen.

From an economic perspective, the SE-HPLC method offers some cost savings. As noted in our analysis, the per-sample cost of our method is approximately $75, a fraction of the cost of an outsourced ELISA ($1200–$2500) or even an in-house ELISA ($400–$600). By leveraging existing HPLC infrastructure, the capital investment required to implement this method is minimal. Furthermore, the combined speed and simplicity of the assay lowers the barrier to entry for academic labs and small biotechnology companies seeking to translate novel biologics into the clinic.

Our study is limited by performing the studies in a single laboratory, and an inter-laboratory comparison would be necessary to establish its reproducibility and facilitate broader adoption. Furthermore, while we validated our method against an ELISA standard, a direct, head-to-head comparison with a cell-based Lindmo assay for the same radiolabeled compound was not performed.

## Conclusion

Use of SE-HPLC for measurement of binding fraction is a simple, specific, and reliable cell-free approach for analyzing the binding interactions between labeled biologics and their respective antigens using existing equipment available in most cGMP-compliant labeling facilities. We demonstrate that this approach is compatible with both fluorescently and radiolabeled biologics, an advantage not offered by any other current assay, enabling streamlined quality control while maintaining compliance with current cGMP guidelines.

## Methods

### General

All chemicals were obtained from commercial suppliers and were of ACS or USP grade (Millipore-Sigma, USA). Size-exclusion chromatography columns were acquired from Cytiva, Waters, or Phenomenex and used according to the manufacturer’s specifications. Fluorescently labeled antibodies, including panitumumab-IRDye800CW and nivolumab-IRDye800CW, were prepared in-house. Radiolabeled biologics, including [89Zr]Panitumumab, [^18^F]GEH200521, and [^18^F]NOTA-ABY-030, were synthesized using in-house procedures in accordance with the appropriate INDs. Radiolabeling precursors and reference standards were sourced externally (GE Healthcare, Almac Sciences) or prepared in-house. Antigens were sources from commercial sources (Sino Biologic or R&D Systems) or provided by GE Healthcare.

### Mobile phase solution

A. Mobile phase A was prepared by mixing Potassium Phosphate (200 mM) and potassium chloride (250 mM) at a pH of 6.2. The solution was filtered through a 0.45 µm nylon membrane to remove any gases. The solution was transferred to the solvent reservoir. This solution was used as the mobile phase for the Biozen 1.8 μm dSEC-2, 200 Å column defined below.
B. Mobile phase B was prepared by mixing sterile water, phosphate-buffered saline (0.05× PBS), sodium azide (10 mM), and sodium chloride (150 mM). The solution was filtered through a 0.45 µm nylon membrane to remove any gases. The solution was transferred to the solvent reservoir. This solution was used as the mobile phase for the Superdex Column defined below.

### Columns and HPLC

An Agilent 1260 Infinity II LC System or a Lablogic LOGI-Chrom system was used to collect data. The HPLC system was equipped with a diode array or multi-wavelength UV detector and a gamma detector. Two different columns were utilized to identify the best separation of the compounds.

A. A Biozen 1.8 μm dSEC-2, 200 Å column (Phenomenex, Part No. 00H-4787-E0) composed of silica was used to test the separation of the panitumumab-IRDye800 peak from the panitumumab-IRDye800-EGFR complex. The column was primed with mobile phase A, described above. Then, 10 µL of the sample was injected onto the column. The flow rate was set to 0.35 mL/min at 25 °C.
B. A Superdex 200 Increase 10/300 GL (Cytiva, Part No. 28990944) was also used to test the separation of the antibody-antigen complex from the unbound antibody. The column was primed with mobile phase B described above, and 20 µL of the sample was injected onto the column. The flow rate was 0.8 mL/min at 25 °C.
C. A Waters Acquity Protein BEH SEC was used for [18F]GEH200521. The column was primed and eluted with a proprietary mobile phase and under proprietary conditions.

HPLC chromatograms were collected, with detection at 280 nM, and either 780 nM (optical) or gamma detection. The peaks of interest are identified and quantified using the area under each peak and assessed as a percentage of the total peak area on the 780 nm/gamma chromatograms.

### Binding assessment of radiolabeled and fluorescently labeled biologics

To assess the binding fraction of labeled biologics, both radiolabeled and fluorescently labeled compounds were incubated with excess antigen and analyzed by size-exclusion high-performance liquid chromatography (SE-HPLC). Analytes included radiolabeled agents such as [^18^F]GEH200521, a CD8-targeting nanobody, and [^18^F]NOYA-ABY-030, an EGFR-targeting affibody, as well as fluorescently labeled antibodies Panitumumab-IRDye800 (pan800), an anti-EGFR antibody, and Nivolumab-IRDye800 (nivo800), a PD-1-targeting antibody.

The antigens used include EGFR (Sino Biological Inc, Cat. #: 10001-H08H-UE), PD-1 (Bio-Techne R&D system, Cat #: 8986-PD-100), CD8 (provided by GEHC), PDL1 (Sino Biological Inc, Cat. #: 10084-H08H), and HER2 (R&D Systems, Cat. #: 1129-ER). The lyophilized antigens were reconstituted according to the manufacturers’ instructions.

For the optimized assay, the labeled compound (5 μL for optical agents, 100 μL for radiolabeled agents) was combined with its corresponding antigen in a 1 mL Eppendorf tube using a pipette and vortexed for 3–5 s at 20–25 °C (Concentrations and antigen amounts described in Table S1). The sample was then transferred into an HPLC vial and injected onto a primed size-exclusion column. Total sample preparation time was approximately 5 min, followed by a 20 or 30-min HPLC run. Detection was performed at 280 nm, and gamma or fluorescence channels, as applicable, based on labeling modality. The overall assay design and methodology are depicted in Fig. 1. The general timeline of the assays discussed in this paper is represented in Fig. 2 (wash steps have been removed, and the simplest version of the assay is assumed).

## Supplementary Information

Below is the link to the electronic supplementary material.

Supplementary Material 1.

## Data Availability

All data generated or analyzed during this study are included in this published article [and its supplementary information files].
