# Supplementary material for: Rapid assessment of target-binding fractions in molecular imaging agents using size-exclusion HPLC
Source: EJNMMI Radiopharm Chem. 2026 Jul 4;11:71. doi: 10.1186/s41181-026-00469-5 (PMC13617156; doi:10.1186/s41181-026-00469-5)
Supplement: Supplementary file 1 [file 41181_2026_469_MOESM1_ESM.docx]

**Supplementary Material**

**Rapid Assessment of Target-Binding Fractions in Theranostic and Imaging Agents Using Size-Exclusion HPLC**

Ashtyn McAdoo^1,2,3^; Kamal Jouad^3^; Eben Rosenthal^2,3^; Adam J. Rosenberg^3,4^*

1. Chemical and Physical Biology, Vanderbilt University, Nashville, TN USA
2. Department of Otolaryngology-Head and Neck Surgery, Vanderbilt University Medical Center, Nashville, TN, USA
3. Vanderbilt University Institute of Imaging Science, Vanderbilt University Medical Center, Nashville, TN USA
4. Department of Radiology and Radiological Sciences, Vanderbilt University Medical Center, Nashville, TN, USA

*****	Corresponding Author: Adam J. Rosenberg, [adam.j.rosenberg@vumc.org](mailto:adam.j.rosenberg@vumc.org), Vanderbilt University Medical Center, Nashville TN, USA

**Panitumumab-IRDye800 – EGFR Binding**

**Figure S1:** SEC of Panitumumab-IRDye800CW (Top, UV @ 280 nm; Bottom UV @ 780 nm)

**Figure S2:** SEC of Panitumumab-IRDye800CW/EGFR Binding Assay (Top, UV @ 280 nm; Bottom UV @ 780 nm)

**Nivolumab-IRDye800 – PD-1 Binding**

**Figure S3:** SEC of Nivolumab-IRDye800CW (Top, UV @ 280 nm; Bottom UV @ 780 nm)

**Figure S4:** SEC of Nivolumab-IRDye800CW/PD-1 Binding Assay (Top, UV @ 280 nm; Bottom UV @ 780 nm)

**[18F]GEH200521 CD8 Binding**

**Figure S5:** SEC of [18F]GEH200521 (Gamma Detector)

**Figure S6:** SEC of [18F]GEH200521 (UV @ 280 nm)

**Figure S7:** SEC of [18F]GEH200521/CD8 Complex (Gamma Detector)

**Figure S8:** SEC of [18F]GEH200521/CD8 Complex (UV @ 280 nm)

**[18F]NOTA-ABY-030**

**Figure S9:** SEC of [18F]NOTA-ABY-030 (Gamma Detector)

**Figure S10:** SEC of [18F]NOTA-ABY-030/EGFR Complex (Gamma Detector)

**Table S1:** Amounts of imaging agents, and antigens used in the described assays:

| Imaging Agent | Concentration | Antigen | Antigen Amount |
| --- | --- | --- | --- |
| Pan800 | 5.00 ± 0.5 mg/mL | EGFR | 100 μg |
| Nivo800 | 2.50 ± 0.5 mg/mL | PD-1 | 100 μg |
| [18F]GEH200521 | 71.2 ± 13.8 μg/mL | CD8α | 200 μg |
| [18F]NOTA-ABY-030 | 11.7 ± 0.5 μg/mL | EGFR | 100 μg |
